# Submucosal metastatic leiomyosarcoma of the colon presenting with intussusception and lower gastrointestinal bleeding: a case report

**DOI:** 10.3389/fonc.2026.1748739

**Published:** 2026-02-03

**Authors:** Honghai Li, Zunfang Yu, Minfeng Ye, Qijing Jin

**Affiliations:** 1 Department of Gastrointestinal Surgery, The First Affiliated Hospital, Shaoxing University, Shaoxing, Zhejiang, China; 2 School of Medicine, Shaoxing University, Shaoxing, Zhejiang, China; 3 Department of Physical Examination Center, Affiliated Hospital of Binzhou Medical University, Binzhou, Shandong, China; 4 Department of Ultrasound Medicine, The First Affiliated Hospital, Shaoxing University, Shaoxing, Zhejiang, China

**Keywords:** colonic, intussusception, leiomyosarcoma, lower gastrointestinal bleeding, metastasis

## Abstract

Colonic leiomyosarcoma (LMS) is a highly aggressive tumor arising from smooth muscle cells and is generally associated with a poor prognosis. This report presents a case of colonic LMS originating from the submucosal layer of the ascending colon. A 76-year-old woman was admitted with intermittent hematochezia persisting for over a month. Imaging studies revealed a tumor in the ascending colon accompanied by intussusception. The patient underwent a right hemicolectomy with systematic lymphadenectomy involving the middle colic root and right branch regions. Postoperative histopathological analysis confirmed colonic LMS arising from the submucosa, with no evidence of lymph node metastasis. Colonic intussusception secondary to LMS is extremely rare, and, to our knowledge, no previous cases of colonic intussusception caused by metastatic sarcoma have been reported. It remains uncertain whether the tumor originated primarily in the colon or resulted from hematogenous metastasis to the colonic mucosa.

## 1 Introduction

Colorectal cancer (CRC) is currently among the most prevalent malignancies in both male and female patients. The majority of colorectal tumors are adenocarcinomas, whereas sarcomas account for only approximately 0.08% of all CRC cases. However, given that most relevant case reports involve single cases or small case series, accurately determining the true incidence remains difficult (1). Although there is currently no standardized treatment protocol for leiomyosarcoma, clinical data analysis suggest that this tumor exhibits poorly responsive to radiotherapy and chemotherapy. Complete surgical resection remains the only effective treatment. Early detection and timely surgical intervention can significantly improve prognosis and overall survival (2). Therefore, early diagnosis and prompt surgery remain critical in the management of leiomyosarcoma.

Intussusception is more common in children, with adult cases accounting for only about 5% of all occurrences. In adults, intussusception represents approximately 1% to 5% of all cases of intestinal obstruction. It occurs more frequently in the small intestine, particularly in the ileocecal region, where 50% to 75% of cases are caused by benign lesions such as Meckel’s diverticulum, postoperative adhesions, or benign tumors (3–5). Intussusception due to leiomyosarcoma is extremely rare, and the few cases reported to date have all involved primary colonic leiomyosarcoma (6–8).

## 2 Case report

A 76-year-old female patient was admitted to the hospital with a chief complaint of intermittent dark red bloody stools lasting for over one month. Her medical history included hypertension, cerebral infarction, a malignant smooth muscle tumor of the right lower extremity, and secondary smooth muscle sarcoma of the right lung.

**Figure 1:**
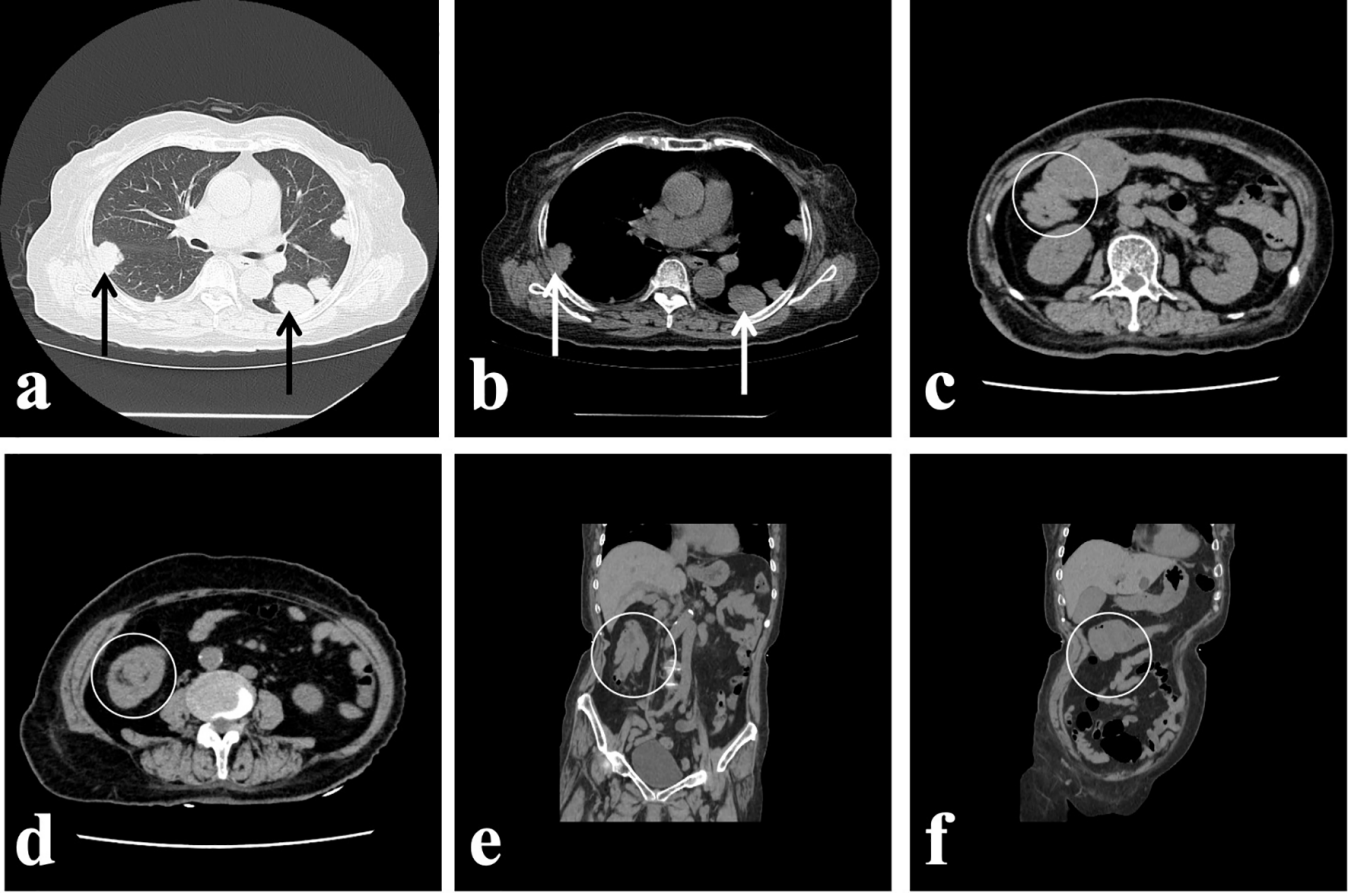
**(a, b)** Multiple masses and nodules are visible in both lungs, raising suspicion for metastatic tumors. **(c)** A soft tissue mass is observed on the posterior wall of the proximal transverse colon near the hepatic flexure, measuring approximately 60 × 40 mm. The intestinal lumen is narrowed, with no enlarged lymph nodes identified surrounding the intestinal wall. **(d)** Intussusception is noted in the ascending colon, characterized by the classic “concentric circle” sign on imaging. **(e)** In the coronal view obtained via multiplanar reconstruction (MPR), the ascending colon appears displaced upward, with the proximal intestinal segment telescoping into the distal ascending colon, forming a characteristic “comet-tail” sign. **(f)** The oblique coronal MPR image reveals the mass located on the lower wall of the transverse colon. It is preliminarily considered an interstitial neoplastic tumor arising from the intestinal wall. Intestinal stenosis is also observed superior to the mass.

One month prior to admission, the patient experienced intermittent episodes of dark red bloody stools without an identifiable cause. These episodes were accompanied with nausea but no vomiting. She also reported a loss of appetite but denied palpitations, cold sweats, abdominal pain, or bloating. Her symptoms improved temporarily following red blood cell transfusions and supportive fluid therapy at another hospital. Because of allergy to iodine-based contrast agents, a non-contrast abdominal CT scan was performed, which revealed marked thickening of the colonic wall (**Figures 1C, F**) at the hepatic flexure, suggestive of a possible neoplastic lesion, along with intussusception sign (**Figures 1D, E**) in the ascending colon. Multiple pulmonary nodules (**Figures 1A, B**) were also observed, raising suspicion for metastatic disease. On physical examination, the patient appeared anemic without cervical lymphadenopathy. The abdomen was soft, with a palpable, firm, mobile, and non-tender mass approximately 5 cm in diameter in the right abdomen, without rebound tenderness. Laboratory tests showed positive fecal occult blood (3+), while tumor markers, including CEA and CA19-9, were within normal limits.

Laparoscopic exploration revealed a lesion near the hepatic flexure of the colon with intussusception. No obvious metastatic nodules were observed in the liver, spleen, or peritoneal cavity, and no significantly enlarged lymph nodes were found at the root of the ileocolic vessels or around the superior mesenteric artery.

**Figure 2:**
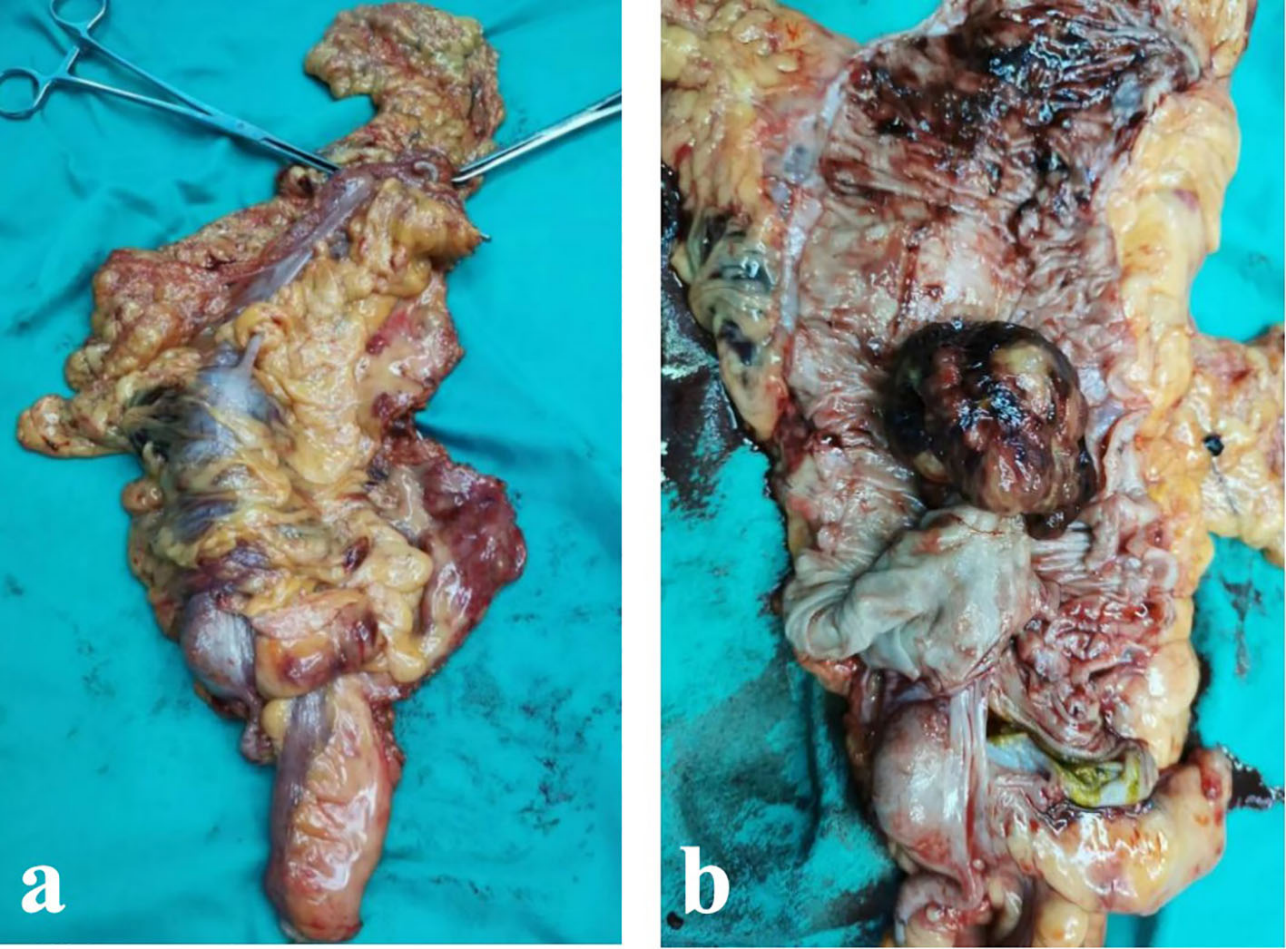
**(a)** A protuberant mass was identified on the colonic mucosa. **(b)** The tumor exhibited a thin pedicle and was confined to the mucosal layer, measuring 5.5 × 5 × 4 cm. Its surface was covered with extensive blood crusts. On sectioning, the cut surface appeared grayish-white to grayish-red with a glossy appearance.

**Figure 3:**
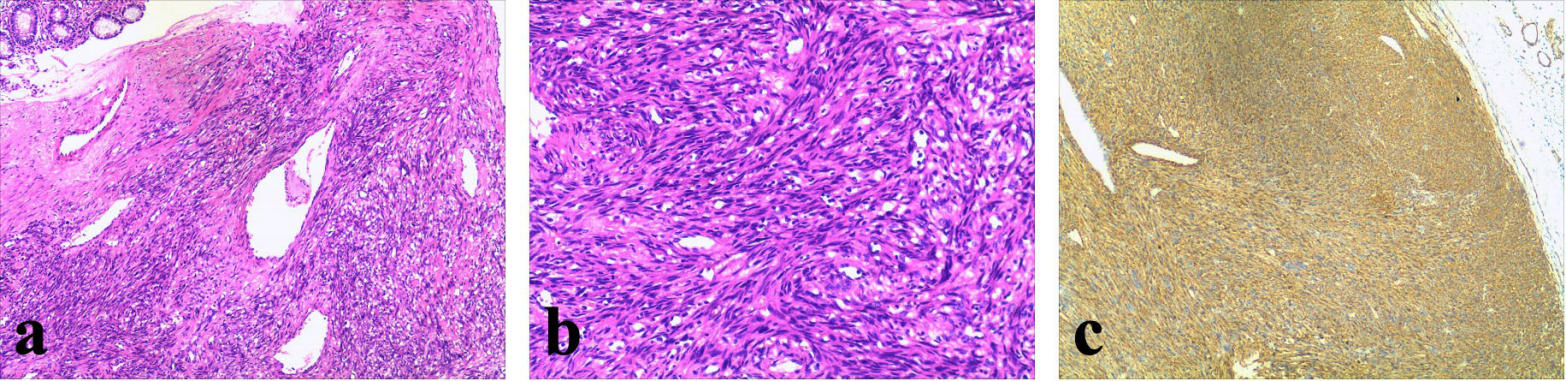
**(a, b)** The tumor was located in the submucosa, with ulceration observed on the mucosal surface. Tumor cells were densely arranged in fascicular or interlacing patterns. Microcystic structures were present within the lesion, and focal areas of hemorrhage were noted. The tumor cells were spindle- or oval-shaped, with eosinophilic cytoplasm, elongated nuclei that were bluntly rounded at both ends, and exhibited marked nuclear pleomorphism with hyperchromasia. Frequent mitotic figures were observed (14 per 10 high-power fields, HPF). **(c)** Immunohistochemical staining showed tumor cells were positive for smooth muscle actin (SMA) and negative for CD117, DOG1, CD34, and broad-spectrum cytokeratin (CK).

Partial right hemicolectomy and ileocolic anastomosis were performed, and the roots of the middle colic artery and the right branch of lymph nodes were dissected. Gross pathological examination (**Figures 2A, B**) revealed a well-circumscribed solid tumor covered by extensive blood crusts, measuring 5.5 × 5 × 4 cm. The wall of the colon extends on the surface of the tumor next to the white tumor. Cross-sectional analysis of the mass showed partial necrosis, cystic changes, and fluid accumulation. On postoperative day five, the patient was able to tolerate porridge without experiencing abdominal pain, distension, or melena. Postoperative pathology confirmed the diagnosis of leiomyosarcoma, which microscopic examination (**Figures 3A, B**) showed that the tumor was located in the submucosa with ulceration on the mucosal surface. The tumor cells were spindle-shaped or ovoid, with eosinophilic cytoplasm and long nuclei, both ends were obtuse and round, and were significantly hyperchromatic. Immunohistochemistry showed positive staining for α-smooth muscle actin (α-SMA). (**Figure 3C**) The patient was discharged uneventfully on the seventh postoperative day.

## 3 Discussion

Leiomyosarcoma (LMS) is a rare malignant mesenchymal neoplasm that primarily originates from smooth muscle cells or their precursors (1), accounting for approximately 10%–20% of all soft tissue sarcomas. About 5%–10% of LMS cases arise from the smooth muscle cells of small vessel walls, the gastrointestinal tract, or uterine tissue. Colonic LMS represents less than 1% of all colorectal malignancies (9), with the stomach being the most common site, followed by the small intestine, colon, and rectum.

The diagnostic and evaluation of gastrointestinal leiomyosarcoma (LMS) remains a significant clinical challenge due to the nonspecific nature of both its clinical manifestations and imaging findings. Common symptoms include abdominal pain, rectal bleeding, intra-abdominal hemorrhage, weight loss, constipation, diarrhea, bowel obstruction, urinary urgency, fever, or, in some cases, no symptoms at all. Computed tomography (CT) similarly lacks pathognomonic features, it remains crucial for assessing tumor size, anatomical location, and potential metastatic involvement. Characteristic imaging findings often include lobulated masses with heterogeneous density, irregular borders, diameters exceeding 5 cm, and central necrosis (10). Visual inspection typically reveals LMS rubbery, poorly demarcated lesions with areas of hemorrhage, necrosis, and cystic degeneration (11). The overlap in clinical and imaging features frequently leads to diagnostic confusion between LMS and gastrointestinal stromal tumors (GISTs). Notably, there are no specific radiological features that definitively confirm LMS, and its vague presentation often results in misdiagnosis as other neoplasms such as GIST.

Soft-tissue smooth-muscle tumors are generally classified into two categories: benign leiomyomas and malignant leiomyosarcomas. Pathological examination remains the gold standard for distinguishing between these tumor types. Key histopathological criteria include nuclear atypia, aggressive malignancy, with mitotic activity serving as a critical predictor of local recurrence and distant metastasis, particularly to well-vascularized organs such as the lungs, liver, and retroperitoneum. Immunohistochemical analysis is essential for differentiating GISTs from other mesenchymal neoplasms. Leiomyosarcomas typically exhibit positive staining for α-smooth muscle actin (α-SMA), desmin, and h-caldesmon, while negative staining for CD34 and S-100 protein helps exclude GISTs and neurogenic tumors. To date, no specific tumor markers have been identified for colonic leiomyosarcoma.

The patient was admitted with recurrent lower gastrointestinal bleeding. However, an allergy to iodine-based contrast agents precluded the use of contrast-enhanced abdominal CT imaging. Furthermore, the presence of intussusception made colonoscopy unsuitable for her so that the pathological results of the tumor could not be obtained. Based on imaging manifestations, ascending colon adenocarcinoma with intussusception and bleeding was initially suspected. Laparoscopic exploration, favored for its minimally invasive nature, was performed instead of conventional laparotomy. Postoperative pathological examination confirmed a leiomyosarcoma of the ascending colon located in the submucosal layer, with no evidence of lymph node metastasis. However, it remains unclear whether the tumor originated primarily in the colon or represented metastasis from a leiomyosarcoma at another site.

This case involved a solitary intraluminal tumor without evident lymph node involvement. Given the patient’s history of leiomyosarcoma in the right lower extremity, metastatic leiomyosarcoma to the colon was considered. Notably, leiomyosarcoma frequently metastasizes to highly vascularized organs, such as the lungs and liver, during advanced stages of disease (12, 13). Metastasis to the colon, particularly with intraluminal growth confined to the mucosal layer, is exceedingly rare. Patients with distant metastases generally have a poor prognosis. Treatment for advanced or metastatic leiomyosarcoma is primarily palliative, aiming to relieve symptoms, prolong survival, and improve quality of life. In this case, the presence of multiple metastases in the lungs and colon indicated advanced-stage disease. Therefore, the primary goal of surgical intervention was to alleviate local symptoms and improve the patient’s quality of life.

For patients suspected of having lower gastrointestinal bleeding, colonoscopy is typically the preferred diagnostic modality. However, it is absolutely contraindicated in cases of hemodynamic instability or hemorrhagic shock (14). In this case, the patient presented with recurrent episodes of bloody stools, indicating active gastrointestinal bleeding, and imaging revealed a tumor complicated by intussusception. Consequently, laparoscopic exploration was deemed the most appropriate diagnostic and therapeutic approach.

## Data Availability

The datasets presented in this study can be found in online repositories. The names of the repository/repositories and accession number(s) can be found in the article/supplementary material.
